# Life Kinetics Training as a Multimodal Neurocognitive Intervention: Enhances Cognitive and Motor Performance in Badminton Athletes

**DOI:** 10.3390/life16050836

**Published:** 2026-05-19

**Authors:** Qonidah Salsabila Senja, Wei Shan, Xindong Ma, Qing Yi, Hongwei Yan

**Affiliations:** 1 Sport Coaching College, Beijing Sport University, Beijing 100084, China; 2 Division of Sports Science and Physical Education, Tsinghua University, Beijing 100084, China; 3 Physical Education, Health and Recreation, Universitas Pendidikan Indonesia, Bandung 40154, Indonesia; 4 China Institute of Sport and Health Science, Beijing Sport University, Beijing 100084, China; 5 Laboratory of Sport Stress and Adaptation of General Administration of Sport, Beijing Sport University, Beijing 100084, China

**Keywords:** life kinetics, neurocognitive training, badminton, brainwave activity, cognitive, motor performance

## Abstract

Background: Life Kinetics training, a multimodal intervention integrating coordinated motor tasks with cognitive challenges, has been proposed as a method to enhance neurocognitive and sport-specific performance. This study examined the effects of an 18-session Life Kinetics program on concentration, working memory, and service performance in junior and intermediate badminton athletes. Methods: Forty athletes (20 junior, 20 intermediate) were included in the final analysis following random allocation to Life Kinetics or control groups. The intervention group performed Life Kinetics training in addition to regular badminton practice for six weeks, while the control group maintained standard training. Outcomes included concentration (Concentration Grid Test), working memory (Digit Span Test), and short- and long-service performance. Data were analyzed using mixed-design ANOVA to assess Group × Time (pre–post) effects, with statistical significance set at *p* < 0.05. Results: Significant Group × Time interaction effects were observed for concentration (F(1, 38) = 152.40, *p* < 0.001, ηp^2^ = 0.80), working memory (F(1, 38) = 168.70, *p* < 0.001, ηp^2^ = 0.82), short-service performance (F(1, 38) = 181.20, *p* < 0.001, ηp^2^ = 0.83), and long-service performance (F(1, 38) = 210.50, *p* < 0.001, ηp^2^ = 0.85). The Life Kinetics group demonstrated substantial improvements across all outcomes, whereas the control group showed minimal changes. Improvements were observed in both junior and intermediate athletes, with variations across outcomes. Exploratory EEG observations suggested a shift toward higher-frequency spectral activity following the intervention. Conclusions: Life Kinetics training was associated with significant improvements in cognitive function and service performance in badminton athletes. These findings support the integration of cognitive–motor training into athlete development programs. However, EEG findings should be interpreted cautiously due to methodological limitations, and future research using larger samples and advanced neuroimaging techniques is warranted.

## 1. Introduction

Badminton is a high-speed racket sport that requires the continuous integration of perceptual information, cognitive processing, and precise motor execution. Among its technical components, the serve plays a critical role in initiating tactical advantage and shaping rally development, particularly during youth and developmental stages [1,2]. Existing research indicates that serving performance in badminton depends not only on technical proficiency and physical capacities but also on cognitive functions, especially concentration, which are essential for information selection, movement planning, and accurate motor execution [3,4,5]. However, in practical training and coaching contexts, traditional badminton training programs predominantly emphasize repetitive technical drills and physical conditioning, often overlooking the systematic development of cognitive components [6,7,8]. In the context of high-speed and dynamically changing competition environments, training approaches that focus solely on motor repetition may be insufficient to meet athletes’ demands for effective cognitive–motor coordination [9,10,11]. Consequently, identifying training interventions that integrate cognitive and motor components has become an important direction for enhancing overall badminton performance. One training approach that explicitly targets cognitive–motor integration is Life Kinetics, a multimodal cognitive–motor training method developed by Horst Lutz [12]. Life Kinetics combines coordinative motor tasks with concurrent cognitive challenges, such as attention shifting, memory processing, and perceptual discrimination, with the aim of activating brain function and promoting neuroplasticity [13,14,15,16]. Several Indonesian studies have described brain jogging as a Life Kinetics based training approach originating from Germany and attributed it to the work of Horst Lutz [7,14,16]. This reflects local terminology usage, as both brain jogging and Life Kinetics are conceptually rooted in training principles developed by Horst Lutz. However, in the international literature, this training approach is most commonly referred to as Life Kinetics. Consistent with this framework, previous research has demonstrated that Life Kinetics or similar cognitively demanding coordination-based training programs can improve both cognitive and motor performance across various sports, including football and fencing [17,18]. In addition, such training approaches have been shown to positively influence working memory in adolescent populations [19]. In recent years, Life Kinetics training has also been preliminarily applied in badminton. Empirical studies have reported that Life Kinetics interventions may improve selected badminton-specific skills, such as overhead lob accuracy [20] as well as serve performance and concentration-related outcomes in children aged 10–12 years [19]. However, the findings across studies are mixed. For instance, other research has reported no significant improvements in certain coordinative abilities following Life Kinetics training in elite youth badminton players [21]. These mixed results suggest that the effects of Life Kinetics training in badminton may depend on factors such as training design, outcome measures, and athlete characteristics, and therefore warrant further systematic investigation [22,23].

Electroencephalography (EEG) studies indicate that elite athletes exhibit distinct neural activity patterns during the execution of motor tasks, such as increased theta-band activity associated with decision-making and alpha-band suppression related to focused concentration [24,25,26]. Gamma-band activity has been closely linked to working memory integration and rapid information processing [27,28]. Life Kinetics training is believed to optimize cognitive–motor connectivity by modulating these neural oscillatory activities [29]. However, research examining its application in badminton athletes, particularly studies investigating its effects and underlying mechanisms from a neurophysiological perspective, remains limited.

To resolve those limitations, the present study aimed to examine the effects of a six-week Life Kinetics training program on concentration, memory, and serve accuracy in junior and intermediate badminton athletes. In addition, EEG measures were employed to explore potential neural mechanisms underlying training-related changes. The following hypotheses were proposed: (1) Life Kinetics training would significantly enhance athletes’ concentration and memory performance, with exploratory EEG measures used to describe potential changes in spectral activity; (2) improvements in cognitive performance will be accompanied by enhanced short-serve and long-serve accuracy.

## 2. Materials and Methods

### 2.1. Sample Size

The sample size was determined using an a priori power analysis conducted with G*Power software (version 3.1.9.7) [30]. The analysis was based on detecting between-group differences in service performance, which was defined as a primary badminton-specific behavioral outcome, as well as related measures of cognitive function. The assumed effect size (Cohen’s d = 0.44) was derived from previous cognitive–motor intervention studies reporting moderate improvements in behavioral and cognitive performance following Life Kinetics and related coordination-based training programs [31]. This effect size was selected to represent a conservative and realistic magnitude of change, consistent with the variability typically observed in human motor and cognitive performance studies. A standard deviation of 2.25, consistent with variability reported in comparable performance measures, was applied in the calculation. With a significance level of α = 0.05 and a statistical power of 0.80, the estimated minimum required sample size was 45 participants. To account for an anticipated dropout rate of approximately 20%, an additional nine participants were recruited, resulting in a total of 54 athletes initially enrolled in the study. Fifty-four badminton athletes were initially enrolled and randomized to either the Life Kinetics training group or the control group. After applying the inclusion and exclusion criteria and accounting for withdrawals, 40 athletes completed the study and were included in the final analysis. The final analyzed sample (*n* = 40) was slightly below the estimated minimum requirement (*n* = 45), which may have reduced statistical power, particularly for detecting smaller effects. Therefore, the reduced sample size may have limited the ability to detect smaller effects and is therefore acknowledged as a limitation.

### 2.2. Randomization

**Figure 1:**
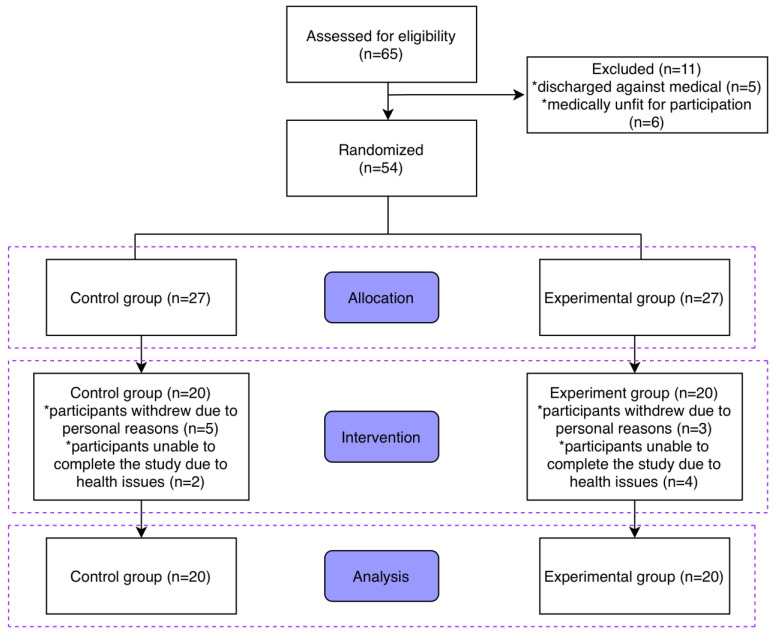
Flow diagram of the trial. * indicates additional explanatory information or special conditions.

This study was conducted as a randomized controlled trial following the CONSORT guidelines [32] (Figure 1). A total of 54 badminton athletes (27 junior and 27 intermediate) were enrolled and randomly assigned to either the Life Kinetics training group or the control group. Participants were first categorized according to competitive level (junior vs. intermediate) and randomization was performed by an independent third party using a computer-generated sequence with a fixed block size of four and a 1:1 allocation ratio, without stratification [33]. Allocation concealment was maintained using sequentially numbered, sealed, and opaque envelopes in accordance with recommended procedures [34]. Due to the nature of the intervention, blinding of coaches and participants was not feasible; however, outcome assessments were performed by evaluators who remained blinded to group allocation to reduce measurement bias [34,35]. All randomization procedures were documented, and after completing all assessments, an independent statistician conducted the final unblinding and performed the statistical analyses.

### 2.3. Participants

A total of 40 badminton athletes completed the study and were included in the final analysis. This sample consisted of 20 junior athletes (10 assigned to the experimental group and 10 to the control group) and 20 intermediate athletes (10 experimental and 10 control). Athletes were recruited from Student Activity Unit, Indonesia University of Education. Recruitment was conducted through coach referrals and training-center announcements. Interested athletes were screened for eligibility by the research team prior to enrollment. Athletes were categorized as junior or intermediate based on their competitive experience and training history within the university badminton program. Junior athletes had between 1 and 3 years of structured badminton training and limited competitive experience at the regional level, whereas intermediate athletes had more than three years of systematic training and regular participation in university or regional competitions.

Inclusion Criteria: Eligible participants were junior and intermediate badminton athletes aged 18–23 years, in good general health, and with no history of neurological, cardiovascular, or musculoskeletal disorders. All athletes engaged in structured badminton training at least three times per week, with each session lasting a minimum of two hours and including technical drills (footwork, stroke mechanics, serving), tactical training, match simulation, and strength or conditioning sessions supervised by certified coaches. Additional criteria included: no high-intensity exercise within the 24 h preceding testing, no lower-limb injuries within the previous six months, and the ability to complete all test procedures safely.

Exclusion Criteria: Athletes were excluded if they experienced a recent lower-limb injury (within six months), engaged in strenuous exercise within 24 h prior to testing, or had any neurological, respiratory, cardiovascular, or hematological conditions that might compromise performance or safety.

Written informed consent was obtained from all participants (and from parents/guardians for minors). The study adhered to the Declaration of Helsinki and was approved by the institutional ethics committee, and was registered in the Indonesia Clinical Trial Registry (INA-06052025427E234).

### 2.4. Training Program

**Figure 2:**
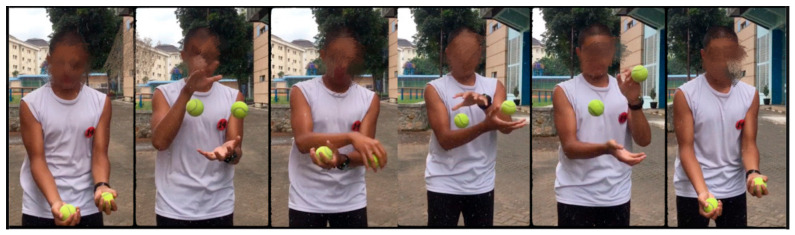
Life Kinetics exercise 1: Juggling training.

**Figure 3:**
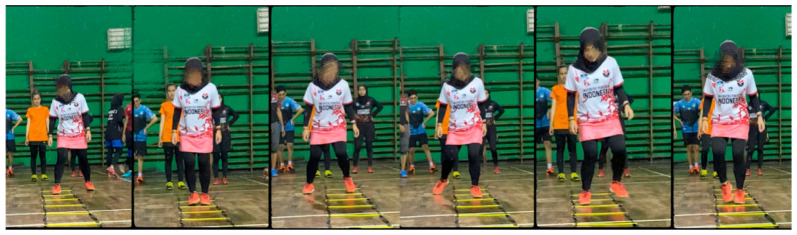
Life Kinetics exercise 2: Agility ladder B2.

The experimental group received a structured Life Kinetics training program conducted three times per week for a total of 18 sessions. Each session lasted 30–60 min and progressed from basic to advanced difficulty levels. Life Kinetics combines physical movement with perceptual and cognitive challenges and has been shown to promote neuroplasticity and enhance cognitive functions such as concentration and memory [36]. Both groups continued to participate in their regular badminton training routines; however, only the experimental group received additional Life Kinetics exercises during the intervention period. The following exercises illustrate the representative Life Kinetics tasks used in this study (Figure 2 and Figure 3).

Juggling was performed individually using two balls. Athletes simultaneously tossed both balls upward and caught them with crossed hands right hand above and left hand below and alternated this pattern continuously. This drill emphasizes hand–eye coordination, bilateral integration, and cognitive–motor coupling by requiring sustained concentration, spatial awareness and rhythmic movement control. The playful, game-like nature of juggling enhances engagement and motivation during training.

The Agility Ladder B2 drill began with the athlete stepping into zigzag-shaped spaces along the ladder using one foot, followed by placing both feet outside the ladder before moving to the next position. This drill trained quick footwork, directional changes, and timing. In the present study, juggling and B2 agility ladder drills were implemented as representative examples of Life Kinetics training, selected to reflect its core principles of cognitive–motor integration while ensuring feasibility and standardization within the intervention period. Task complexity and movement speed were progressively increased across sessions to maintain both cognitive load and physical challenge.

Control Group Training: The control group performed traditional badminton training with an equivalent duration and frequency. Training sessions included standard technical drills (footwork, serving, and stroke mechanics), basic agility exercises, and tactical gameplay. This ensured comparable training volume and intensity between groups while isolating the cognitive–motor effects of the Life Kinetics intervention. The intervention consisted of 18 sessions over six weeks (three sessions per week) and was conducted alongside regular badminton training rather than replacing it.

### 2.5. Training Program Instrument and Test Procedure

This study employed a combination of cognitive tests, badminton skill assessments, and EEG-based measurements to evaluate changes in concentration, working memory, brainwave activity, and service performance following the Life Kinetics intervention. All outcome measures were assessed twice: at baseline (pre-test) before the intervention and again after completion of the training program (post-test), using identical procedures, task order, and testing conditions with EEG recording synchronized across the cognitive and serving tasks to capture neural dynamics associated with cognitive–motor performance.

#### 2.5.1. Cognitive Assessment

Cognitive performance was assessed through two established instruments measuring concentration and working memory. Concentration was evaluated using the Concentration Grid Test adapted from Harris and Harris [37] in which participants located numbers from 00 to 99 within a randomized 10 × 10 grid as quickly and accurately as possible within fixed time period. Performance was quantified as the total number of correctly identified targets, with higher scores indicating better attentional performance. Working memory was measured using the forward and backward Digit Span Test [38] In the forward condition, participants recalled numerical sequences in order, while in the backward condition, they reproduced sequences in reverse, engaging short-term memory storage as well as executive manipulation processes. Forward and backward scores were analyzed separately and then summed to obtain a composite working memory score. Together, these two instruments provided a comprehensive assessment of attentional focus and working memory capabilities relevant to cognitive–motor performance in badminton.

#### 2.5.2. Badminton Skill Assessment

Badminton performance was measured using standardized serving skill assessments commonly applied in sport performance research. Short-service accuracy was evaluated using the Frank M. Verduci Short Service Skill Test [39,40] which examines precision, consistency, and control in low-trajectory serves. Long-service performance was assessed using the James Poole Long Service Skill Test [41]. For each test, athletes performed a predetermined number of service attempts toward designated target zones on the court. Performance was quantified using a score-based system, in which points were awarded for successful placements within target areas, as defined in the original test protocols. Scores from all attempts were summed to yield a total service performance score for each condition, with higher scores reflecting greater service accuracy and consistency. The outcome measures were expressed as unitless performance scores.

#### 2.5.3. EEG Measurement Across Cognitive and Serving Tasks

**Figure 4:**
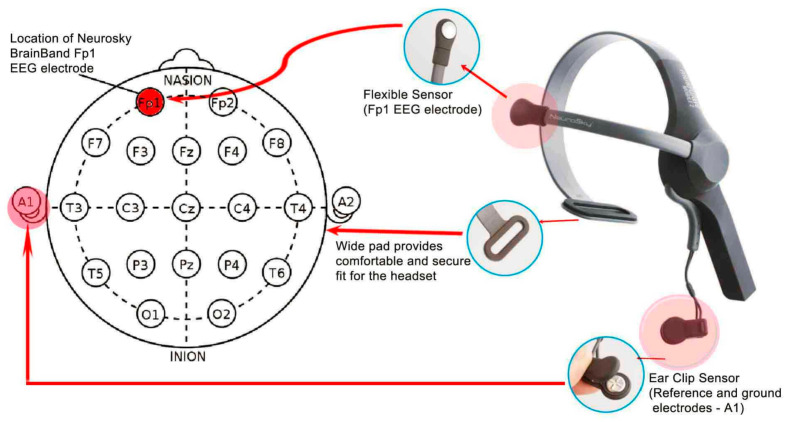
NeuroSky MindWave headset. The red arrows indicate electrode connections/positions, the blue circles highlight headset components, and the red shaded circles indicate the active sensor and reference electrode locations.

To capture neural processes underlying cognitive and motor performance, EEG recordings were collected continuously during the concentration test, memory test, and both serving assessments. Brain activity was recorded using the NeuroSky MindWave headset (NeuroSky Inc., San Jose, CA, USA) (Figure 4), a single-channel EEG device positioned at the frontal region (Fp1), with a sampling rate of 512 Hz. The device transmits data to the WujiBrainwave software 1.2.7 for acquisition and processing [42].

The NeuroSky system applies Fast Fourier Transform (FFT) to decompose raw EEG signals into their constituent frequency components. Based on this transformation, the device provides estimates of spectral activity across standard EEG frequency ranges, including Delta (0.5–4 Hz), Theta (4–8 Hz), Alpha (8–13 Hz), Beta (13–30 Hz), and Gamma (30–50 Hz). These frequency ranges are consistent with commonly accepted EEG band classifications in the literature [27,28,29]. In the present study, EEG outputs were summarized as dominant frequency estimates (Hz), representing the prevailing spectral characteristics of the signal during task performance. EEG data were used as exploratory indicators of relative spectral changes and were intended to complement behavioral outcomes. By synchronizing EEG recording with both cognitive and badminton tasks, the study captured real-time neural markers of attention, memory, and cognitive–motor integration, providing an exploratory description of task-related EEG spectral characteristics during performance.

### 2.6. Data Analysis

EEG signals recorded by the NeuroSky MindWave headset were processed using the WujiBrainwave software, which provides acquisition, visualization, and frequency-domain analysis of the signal. Raw EEG data transmitted via Bluetooth were filtered and smoothed to isolate activity within standard frequency bands, including Delta (0.5–4 Hz), Theta (4–8 Hz), Alpha (8–13 Hz), Beta (13–30 Hz), and Gamma (30–50 Hz). In addition to band power estimates, dominant frequency (Hz) was extracted to summarize overall spectral characteristics as the relative magnitude of spectral energy within each frequency range over time, derived from signal amplitude following spectral transformation. All processed data were exported in numerical form for statistical analysis and integration with behavioral outcomes.

All statistical analyses were conducted using IBM SPSS Statistics version 27.0 (IBM Corp., Armonk, NY, USA). Descriptive data are presented as mean ± standard deviation. Baseline characteristics (Table 1) were compared between junior and intermediate athletes using independent-samples *t*-tests. Data distribution was assessed using the Shapiro–Wilk test, and homogeneity of variance was evaluated using Levene’s test. Primary inferential analyses were performed using mixed-design ANOVA to examine Group (Life Kinetics vs. control) × Time (pre-test vs. post-test) effects on concentration, Digit Span scores, and short- and long-service accuracy. Mixed-design ANOVA was selected as the main analytical approach due to its robustness to moderate deviations from normality in balanced experimental designs. Effect sizes were reported as partial eta squared (ηp^2^). Within-group pre–post changes are presented descriptively to illustrate the magnitude and direction of change and were not used as the primary basis for statistical inference. Following significant Group × Time interactions, Bonferroni-adjusted post hoc comparisons were conducted to examine (1) within-group pre–post differences and (2) between-group differences at baseline and post-test. EEG data were analyzed descriptively to examine changes in dominant frequency values across conditions. Due to methodological limitations, EEG results are interpreted cautiously and primarily as supportive descriptive data. Statistical significance was set at *p* < 0.05 (two-tailed) for all analyses.

## 3. Results

### 3.1. Baseline Characteristics

The baseline characteristics of the study participants, including their demographic, anthropometric, and cognitive profiles, are summarized in Table 1 presents baseline characteristics compared using independent-samples *t*-tests. A detailed comparison of these variables is presented below to illustrate initial similarities and differences between the junior and intermediate groups prior to the intervention. A total of 40 badminton athletes participated in this study, consisting of 20 junior athletes and 20 intermediate athletes. The combined descriptive characteristics of all participants are summarized in Table 1. Across both groups, the athletes had an average age of 20 years (range: 18–22), with a mean height of 167.9 cm and an average body weight of 63.9 kg. This corresponds to a mean BMI of 20.55 kg/m^2^ (range: 19.72–26.57), indicating that the participants were generally within the normal and healthy weight range for competitive badminton athletes. Cognitive ability, assessed using a standardized IQ measure, showed an overall mean score of 105.8, with individual scores ranging from 98 to 123, reflecting normal to above-average cognitive functioning. Independent-samples *t*-tests revealed significant differences between junior and intermediate athletes across all baseline variables (all *p* < 0.05), indicating expected differences in age, anthropometric characteristics, and experience level. In addition, baseline comparisons between the Life Kinetics (LK) and control groups showed no significant differences across all measured variables (all *p* > 0.05), indicating that the groups were comparable prior to the intervention.

Mixed-design ANOVA analyses revealed significant Group × Time interaction effects across all performance variables (Table 2). Significant interactions were observed for concentration (F(1, 38) = 152.40, *p* < 0.001, ηp^2^ = 0.80), memory (F(1, 38) = 168.70, *p* < 0.001, ηp^2^ = 0.82), short-service performance (F(1, 38) = 181.20, *p* < 0.001, ηp^2^ = 0.83), and long-service performance (F(1, 38) = 210.50, *p* < 0.001, ηp^2^ = 0.85), indicating that the Life Kinetics group improved significantly more over time compared to the control group.

Post hoc (follow-up) comparisons were conducted following significant Group × Time interaction effects to further examine within-group and between-group differences across all outcomes (Table 3).

The results demonstrated that the Life Kinetics (LK) group exhibited statistically significant improvements from pre-test to post-test in concentration, working memory, short-service, and long-service performance (all *p* < 0.001). Percentage values reported in this study represent relative changes in behavioral performance scores, calculated as (post − pre)/pre × 100. EEG results, in contrast, are expressed as dominant frequency values (Hz) obtained during task performance and represent spectral characteristics independent of behavioral percentage measures. The control group showed only minimal changes across these variables, and these changes did not reach statistical significance (all *p* > 0.05). Between-group comparisons indicated no substantial baseline differences across most variables (*p* > 0.05), with minor variations accounted for by the Group × Time interaction analysis. At post-test, the LK group demonstrated significantly higher scores than the control group across all performance variables (all *p* < 0.001), indicating a clear effect of the intervention. To enhance transparency and account for individual variability, participant-level change scores (Δ = post − pre) are provided in Supplementary Table S1.

### 3.2. Effect of Life Kinetics Training on Cognitive Function and Motor Performance in Junior Badminton Athletes

Significant Group × Time interaction effects were observed across all performance variables Table 2. The Life Kinetics (LK) group showed marked improvements from pre- to post-test, whereas the control group exhibited only minimal changes Table 4. For example, concentration increased from 5.30 ± 2.16 to 12.70 ± 2.67 in the LK group. Comparable gains were also evident for memory, short-service, and long-service performance.

### 3.3. Effect of Life Kinetics Training on Cognitive Function and Motor Performance in Intermediate Badminton Athletes

A similar pattern was found in intermediate athletes, with significant Group × Time interaction effects across all variables Table 2. The LK group demonstrated substantial increases in concentration, memory, and service performance, while the control group showed only modest improvements Table 5. These findings indicate a consistent advantage of the Life Kinetics intervention across performance outcomes.

### 3.4. EEG Spectral Characteristics in Junior and Intermediate Badminton Athletes

At post-test, the Life Kinetics (LK) group [Table 6] demonstrated substantially higher dominant frequency values across all task conditions compared to the control group. Specifically, dominant frequencies in the LK group ranged from approximately 33.8 to 36.5 Hz across concentration, memory, short-service, and long-service tasks. These values fall within the gamma frequency range (30–50 Hz), which is commonly associated with higher-frequency spectral activity during task engagement. In contrast, the control group remained within a lower range of approximately 16.6 to 18.1 Hz, corresponding to the beta frequency band (13–30 Hz), with minimal deviation from baseline levels. This clear post-intervention separation between groups indicates a consistent shift from beta-range activity at baseline toward higher-frequency (gamma-range) spectral characteristics in the LK group. The magnitude and consistency of this shift across both cognitive and motor tasks suggest a generalized pattern of increased higher-frequency engagement following the intervention, whereas the control group exhibited only negligible changes. Importantly, these post-test findings are consistent with the significant Group × Time interaction effects observed in the inferential analysis, confirming that the divergence between groups emerged specifically after the intervention period. Nevertheless, these results should be interpreted as descriptive indicators of spectral activity derived from single-channel EEG recordings and should not be considered direct evidence of specific neurophysiological mechanisms or localized cortical processes.

**Figure 5:**
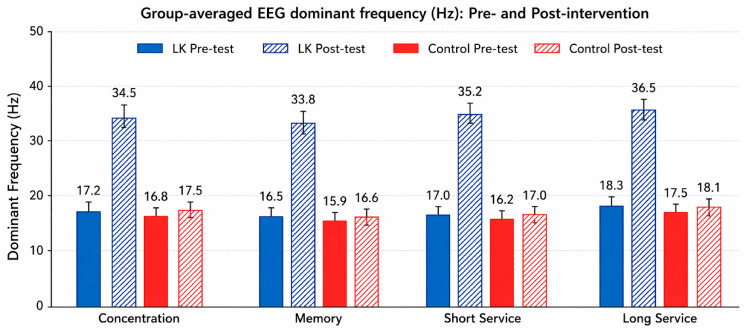
Group-averaged EEG dominant frequency (Hz) before and after the intervention. EEG frequency bands were categorized as follows: Delta (0.5–4 Hz), Theta (4–8 Hz), Alpha (8–13 Hz), Beta (13–30 Hz), and Gamma (30–50 Hz).

Mixed-design ANOVA analysis of EEG dominant frequency values (Figure 5) revealed significant Group × Time interaction effects across all task conditions Table 7, indicating that changes in spectral characteristics differed systematically between the Life Kinetics and control groups over time. Specifically, the Life Kinetics group demonstrated consistent increases in dominant frequency values from pre- to post-test across cognitive and motor task conditions, whereas the control group exhibited only minimal changes. These findings reflect a clear divergence in task-related spectral patterns between groups and provide quantitative support for the observed differences in EEG spectral characteristics following the intervention. Detailed EEG spectral activity maps illustrating pre–post changes are provided in the Supplementary Materials (Figures S1 and S2) for transparency and visualization purposes.

## 4. Discussion

### 4.1. Effects of Life Kinetics Training on Cognitive Function in Junior and Intermediate Athletes

The findings of this study demonstrate that Life Kinetics training produced notable enhancements in cognitive performance among both junior and intermediate badminton athletes. As presented in Table 4, junior athletes exhibited significant improvements in concentration (mean difference = 7.4) and memory (mean difference = 14.4), corresponding to gains of 58% and 63%, respectively. These results are consistent with prior evidence suggesting that younger athletes possess heightened neuroplastic potential, allowing for greater improvements in executive functioning and working memory when exposed to cognitively enriched motor-training environments [16,43].

Intermediate athletes also demonstrated substantial cognitive gains, although their adaptations followed a slightly different pattern. As illustrated in Table 5, concentration improved by 13.9 points (68%), and memory increased by 13 points (56%). While relative improvements were slightly smaller compared with junior athletes, absolute performance gains remained considerable. This pattern aligns with research indicating that athletes with greater perceptual–motor maturity exhibit more efficient attentional control, cue filtering, and working-memory retrieval due to accumulated sport experience [44,45].

EEG observations complemented these behavioral outcomes. In addition to the descriptive patterns observed, statistical analysis of EEG dominant frequency values revealed significant Group × Time interaction effects across all task conditions Table 7, indicating that changes in spectral characteristics differed systematically between the Life Kinetics and control groups over time. As summarized in Table 6, post-intervention recordings demonstrated consistently higher dominant frequency values compared with pre-test conditions in the Life Kinetics group across all task conditions, whereas only minimal changes were observed in the control group. These descriptive patterns are consistent with the prior EEG literature linking beta-band activity to sustained attention and sensorimotor processing [46,47,48]. Collectively, the behavioral findings demonstrate improvements in attentional stability and working memory following Life Kinetics training, which are directly relevant to badminton performance—requiring rapid information processing, anticipation, and precise motor execution.

### 4.2. Influence of Life Kinetics Training on Service Performance

Life Kinetics training also resulted in meaningful improvements in serving performance across skill levels. As shown in Table 5, junior athletes experienced increases of 14.4 points in short service and 16.6 points in long service. These improvements likely reflect enhanced coordination, timing control, and more effective integration of perceptual information during motor execution. Such findings are consistent with previous research highlighting the role of cognitive functions, particularly attention and working memory, in badminton skill performance [49,50].

Intermediate athletes similarly showed substantial improvements, as shown in Table 4, with short service increasing by 11.3 points and long service by 22.4 points. The larger gains in long-service performance may be related to improved control of force, trajectory planning, and decision-making processes, which are influenced by both cognitive and motor experience [51,52].

The cognitive–motor nature of service execution explains these improvements. Serving requires athletes to: (1) regulate attentional focus, (2) retrieve learned motor schemas, (3) anticipate opponent positioning and (4) execute coordinated movement sequences. Life Kinetics directly trains these processes by pairing perceptual load with motor execution, promoting neurocognitive integration crucial to serving efficiency.

### 4.3. Neural Oscillatory Mechanisms Underlying Performance Enhancement

The EEG findings indicate changes in spectral characteristics following Life Kinetics training. In both junior and intermediate athletes, post-intervention recordings consistently showed higher dominant frequency values compared with pre-test conditions, reflecting a systematic shift in task-related spectral activity during performance. These observations are interpreted in relation to the established EEG literature. Beta-band activity has been associated with sustained attention and sensorimotor processing [46,47], whereas higher-frequency activity, including gamma-band oscillations, has been linked to cognitive functions such as working memory, attentional integration, and rapid information processing [48,49,50,51,53]. The spectral patterns observed in the present study are consistent with these general functional associations. Overall, the EEG results characterize global changes in spectral activity during task performance and provide complementary information alongside the behavioral findings. The primary conclusions of the study remain grounded in the statistically validated improvements in cognitive and motor performance [52,54,55,56].

### 4.4. Differential Adaptations Between Junior and Intermediate Athletes

Although both groups benefited from Life Kinetics training, junior athletes demonstrated relatively greater percentage improvements across most variables. This difference may be attributed to heightened neuroplasticity in younger athletes, allowing for more rapid synaptic strengthening and cognitive–motor integration [43].

Intermediate athletes demonstrated greater absolute improvements in long-service performance and concentration control. These findings are consistent with research showing that experienced athletes possess more developed perceptual–attentional frameworks, enabling them to utilize cognitive training more effectively and strategically [44]. Additionally, their more mature sensorimotor systems may allow them to better capitalize on the observed neural adaptations. Overall, these patterns suggest that Life Kinetics represents a scalable intervention: junior athletes benefit through broader developmental gains, while intermediate athletes benefit through more refined, performance-specific cognitive–motor efficiency.

### 4.5. Practical Implications for Badminton Training

The results suggest that Life Kinetics training can serve as a valuable complement to technical badminton instruction. By enhancing concentration, working memory, and motor coordination, Life Kinetics may improve performance consistency, reduce technical errors, and support more effective decision-making during match play. Coaches may integrate Life Kinetics sessions into regular training micro-cycles to enhance cognitive readiness alongside motor execution. This approach aligns with evidence showing that cognitively enriched training environments promote superior learning outcomes and more durable motor skills [55,56].

### 4.6. Limitations

Several limitations should be acknowledged when interpreting the findings of this study. First, although statistically sufficient, the sample sizes for both junior and intermediate groups were relatively small, which may limit the generalizability of the results to wider badminton populations. Larger and more diverse samples including elite or recreational athletes could provide a more comprehensive understanding of the effects of Life Kinetics training. Second, the EEG measurements were obtained using the NeuroSky MindWave system, which utilizes a single electrode and produces algorithmically derived brainwave indices rather than raw multi-channel microvolt recordings. Although this device is appropriate for detecting general patterns of oscillatory activity and relative changes across sessions, the neural mechanisms underlying training-related improvements cannot be mapped in detail. Future studies employing high-density EEG or multimodal neuroimaging methods would allow for more precise characterization of neurocognitive processes associated with Life Kinetics training. Third, the study only assessed pre–post changes immediately following the intervention, with no retention testing to examine the durability of cognitive or motor improvements. Without long-term follow-up, it remains unclear whether the gains in concentration, memory, and service performance persist over time. Fourth, the study focused primarily on service performance, which represents only one technical component of badminton. Other crucial aspects such as footwork speed, tactical anticipation, defensive transitions, and rally performance were not evaluated, limiting the scope of performance-related conclusions.

### 4.7. Future Directions

Future research should address these limitations through several targeted approaches. First, studies involving larger and more diverse athlete samples across age groups and competitive levels are needed to strengthen generalizability. Second, the use of advanced neuroimaging techniques such as high-density EEG, fNIRS, or MEG would allow more precise identification of neural mechanisms underlying cognitive–motor training. Third, longitudinal designs are recommended to determine whether improvements in cognitive and motor performance are maintained over time and to identify optimal training duration and frequency. Fourth, future studies should incorporate a broader range of performance measures, including agility, reaction time, visual search strategies, and decision-making during match play. Finally, individualized or adaptive Life Kinetics programs may further enhance training effectiveness. Tailoring task difficulty to athletes’ cognitive and motor profiles may optimize learning outcomes and reduce overload.

## 5. Conclusions

This study investigated the effects of an 18-session Life Kinetics training program on concentration, working memory, and service performance in junior and intermediate badminton athletes using behavioral assessments and EEG measures. The results demonstrated significant improvements across all cognitive and motor outcomes following the intervention, with increases in concentration, memory, and both short- and long-service accuracy in the Life Kinetics group compared to the control group. Exploratory EEG observations suggested relatively higher dominant frequency values following training, indicating descriptive changes in spectral activity during task performance. Overall, the findings highlight the potential value of incorporating multimodal cognitive–motor training into badminton development programs. Life Kinetics appears to effectively target both cognitive and technical aspects of performance, supporting its application as a complementary training approach. Future research should employ larger samples, advanced multi-channel neuroimaging methods, and longitudinal designs to further clarify underlying mechanisms and assess the long-term sustainability of training effects.

## Figures and Tables

**Table 1:** Baseline characteristics assessment.

Variable	Junior (*n* = 20) Mean ± SD	Intermediate (*n* = 20) Mean ± SD	*p*-Value
Age (years)	18.9 ± 0.74	21.1 ± 0.83	<0.001
Height (cm)	165.8 ± 4.12	170.0 ± 4.98	0.012
Weight (kg)	61.2 ± 5.80	66.6 ± 6.01	0.008
BMI (kg/m^2^)	19.99 ± 1.62	21.11 ± 1.92	0.041
IQ	103.4 ± 4.88	108.2 ± 6.33	0.006

Variables are presented as mean ± standard deviation (SD). *n* = number of subjects in each group. BMI = Body Mass Index. *p*-values are based on independent-samples *t*-tests comparing junior and intermediate groups.

**Table 2:** Mixed-design ANOVA results for performance outcomes.

Outcome	Effect	F(1, 38)	*p*-Value	Partial η^2^
Concentration	Group × Time	152.40	<0.001	0.80
Memory	Group × Time	168.70	<0.001	0.82
Short Service	Group × Time	181.20	<0.001	0.83
Long Service	Group × Time	210.50	<0.001	0.85

Results from mixed-design ANOVA examining Group (Life Kinetics vs. control) × Time (pre vs. post) interactions. ηp^2^ = partial eta squared (effect size). All effects were statistically significant at *p* < 0.001.

**Table 3:** Post hoc comparisons of performance outcomes.

Outcome	Comparison	Mean Difference (Δ)	*p*-Value
Concentration	LK Pre vs. Post	+7.40	<0.001
	Control Pre vs. Post	+0.50	0.180
	LK vs. Control (Pre)	+0.80	0.310
	LK vs. Control (Post)	+7.80	<0.001
Memory	LK Pre vs. Post	+14.40	<0.001
	Control Pre vs. Post	+0.70	0.150
	LK vs. Control (Pre)	−2.50	0.120
	LK vs. Control (Post)	+11.50	<0.001
Short Service	LK Pre vs. Post	+14.30	<0.001
	Control Pre vs. Post	+0.80	0.140
	LK vs. Control (Pre)	+1.10	0.270
	LK vs. Control (Post)	+14.70	<0.001
Long Service	LK Pre vs. Post	+16.60	<0.001
	Control Pre vs. Post	+2.20	0.090
	LK vs. Control (Pre)	+6.00	0.080
	LK vs. Control (Post)	+21.00	<0.001

Post hoc comparisons were conducted using Bonferroni-adjusted pairwise comparisons following significant Group × Time interaction effects in the mixed-design ANOVA. Mean differences represent changes between conditions. To improve transparency and address individual variability, individual participant change scores (Δ = post − pre) for all outcomes are provided in Supplementary Table S1, allowing direct evaluation of within-subject responses to the intervention.

**Table 4:** Performance outcomes of junior badminton athletes.

Outcome	Group	Pre (Mean ± SD)	Post (Mean ± SD)	Δ (Post − Pre)
Concentration	LK	5.30 ± 2.16	12.70 ± 2.67	+7.40
	Control	4.50 ± 0.97	4.90 ± 1.37	+0.40
Memory	LK	8.30 ± 2.41	22.70 ± 3.89	+14.40
	Control	10.80 ± 1.30	11.20 ± 1.03	+0.40
Short Service	LK	5.50 ± 2.07	19.80 ± 2.78	+14.30
	Control	4.40 ± 1.65	5.10 ± 1.10	+0.70
Long Service	LK	17.20 ± 3.62	33.80 ± 2.86	+16.60
	Control	11.20 ± 1.62	12.80 ± 2.86	+1.60

Values are presented as mean ± SD. Δ represents descriptive within-group change (post − pre). Statistical significance was evaluated using mixed-design ANOVA (Group × Time).

**Table 5:** Performance outcomes of intermediate badminton athletes.

Outcome	Group	Pre (Mean ± SD)	Post (Mean ± SD)	Δ (Post − Pre)
Concentration	LK	6.50 ± 1.58	20.40 ± 1.65	+13.90
	Control	10.00 ± 2.67	10.70 ± 2.45	+0.70
Memory	LK	10.10 ± 3.60	23.10 ± 3.54	+13.00
	Control	15.60 ± 1.35	16.40 ± 1.35	+0.80
Short Service	LK	11.60 ± 2.72	22.90 ± 1.91	+11.30
	Control	12.90 ± 1.97	13.40 ± 1.96	+0.50
Long Service	LK	16.50 ± 3.50	38.90 ± 3.99	+22.40
	Control	15.20 ± 2.49	17.80 ± 3.23	+2.60

Values are presented as mean ± SD. Δ represents descriptive within-group change (post − pre). Statistical significance was evaluated using mixed-design ANOVA (Group × Time).

**Table 6:** EEG spectral characteristics (dominant frequency, Hz).

Outcome	Group	Pre-Test (Hz, Mean ± SD)	Post-Test (Hz, Mean ± SD)	Δ (Hz)
Concentration	LK	17.2 ± 1.8	34.5 ± 3.2	+17.3
	Control	16.8 ± 1.5	17.5 ± 1.7	+0.7
Memory	LK	16.5 ± 2.0	33.8 ± 3.5	+17.3
	Control	15.9 ± 1.6	16.6 ± 1.8	+0.7
Short Service	LK	17.0 ± 1.9	35.2 ± 3.1	+18.2
	Control	16.2 ± 1.7	17.0 ± 1.6	+0.8
Long Service	LK	18.3 ± 2.1	36.5 ± 3.6	+18.2
	Control	17.5 ± 1.8	18.1 ± 2.0	+0.6

Group-averaged EEG spectral characteristics expressed as dominant frequency values (Hz) recorded during task performance. Values are presented as mean ± standard deviation (SD) for pre-test and post-test conditions, with Δ indicating descriptive change (post − pre). Values represent group-level averages calculated across all participants within each group, including both junior and intermediate athletes in the control and experiment groups (*n* = 10 per subgroup).

**Table 7:** Mixed-design ANOVA results for EEG dominant frequency (Hz).

Outcome	Effect	F(1, 38)	*p*-Value	Partial η^2^
Concentration (EEG)	Group × Time	165.20	<0.001	0.81
Memory (EEG)	Group × Time	158.40	<0.001	0.80
Short Service (EEG)	Group × Time	172.60	<0.001	0.82
Long Service (EEG)	Group × Time	180.10	<0.001	0.84

Mixed-design ANOVA results examining Group (Life Kinetics vs. control) × Time (pre-test vs. post-test) interaction effects for EEG dominant frequency values (Hz) across task conditions. Partial η^2^ = partial eta squared (effect size). All interaction effects were statistically significant at *p* < 0.001.

## Data Availability

The original contributions presented in this study are included in the article/Supplementary Material. Further inquiries can be directed to the corresponding author.

## Supplementary Materials

The following supporting information can be downloaded at: https://www.mdpi.com/article/10.3390/life16050836/s1, Table S1. Individual pre–post difference scores for concentration, memory, short-service, and long-service performance in all participants; Figure S1. EEG Brainwave Activity and Performance of JBA; Figure S2. EEG Brainwave Activity and Performance of IBA.

## Abbreviations

The following abbreviations are used in this manuscript:

LK	Life Kinetics
EEG	Electroencephalography
JBA	Junior badminton athletes
IBA	Intermediate badminton athletes
