# Supplementary material for: Life Kinetics Training as a Multimodal Neurocognitive Intervention: Enhances Cognitive and Motor Performance in Badminton Athletes
Source: Life (Basel). 2026 May 19;16(5):836. doi: 10.3390/life16050836 (PMC13208401; doi:10.3390/life16050836)
Supplement: Supplementary file 1 [file life-16-00836-s001.zip › life-4210320-supplementary.pdf]

Supplementary Table S1. Individual pre–post difference scores for concentration, memory, short-service, and long-service performance in all participants.

Subject ID	Group	Level	Δ Concentration	Δ Memory	Δ Short Service	Δ Long Service
S01	LK	Junior	+7	+14	+14	+16
S02	LK	Junior	+8	+13	+15	+17
S03	LK	Junior	+6	+15	+13	+16
S04	LK	Junior	+7	+14	+14	+17
S05	LK	Junior	+8	+14	+15	+16
S06	LK	Junior	+7	+15	+14	+17
S07	LK	Junior	+6	+13	+13	+15
S08	LK	Junior	+8	+14	+15	+16
S09	LK	Junior	+7	+14	+14	+16
S10	LK	Junior	+7	+14	+14	+16
S11	Control	Junior	+0	+1	+1	+2
S12	Control	Junior	+1	+0	+0	+1
S13	Control	Junior	+0	+0	+1	+1
S14	Control	Junior	+1	+1	+0	+2
S15	Control	Junior	+0	+0	+1	+1
S16	Control	Junior	+1	+1	+1	+2
S17	Control	Junior	+0	+0	+0	+1
S18	Control	Junior	+1	+1	+1	+2
S19	Control	Junior	+0	+0	+1	+1
S20	Control	Junior	+1	+1	+0	+2
S21	LK	Intermediate	+14	+13	+11	+22
S22	LK	Intermediate	+13	+12	+10	+21
S23	LK	Intermediate	+14	+13	+11	+23
S24	LK	Intermediate	+15	+14	+12	+22
S25	LK	Intermediate	+13	+13	+11	+21
S26	LK	Intermediate	+14	+12	+11	+22
S27	LK	Intermediate	+13	+13	+10	+23
S28	LK	Intermediate	+14	+14	+11	+22
S29	LK	Intermediate	+13	+13	+11	+22
S30	LK	Intermediate	+14	+13	+11	+22
S31	Control	Intermediate	+1	+1	+0	+2
S32	Control	Intermediate	+0	+1	+1	+3
S33	Control	Intermediate	+1	+0	+0	+2
S34	Control	Intermediate	+0	+1	+1	+3
S35	Control	Intermediate	+1	+1	+0	+2
S36	Control	Intermediate	+0	+0	+1	+3
S37	Control	Intermediate	+1	+1	+1	+2
S38	Control	Intermediate	+0	+1	+0	+3
S39	Control	Intermediate	+1	+0	+1	+2
S40	Control	Intermediate	+0	+1	+0	+3

Δ represents the change from pre-test to post-test (Post − Pre). Positive values indicate improvement in performance outcomes.

The individual change scores presented in Table S1 demonstrate a consistent and uniform pattern of improvement within the Life Kinetics (LK) group across all outcome measures. All participants in the LK group exhibited positive gains in concentration, memory, and service performance. In contrast, the control group showed minimal and variable changes, with substantially smaller magnitudes of improvement across all variables. This distribution of individual responses supports the group-level findings and indicates that the observed effects were consistent across participants, rather than driven by a small subset of individuals.

Supplementary Figure S1 EEG Brainwave Activity and Performance of JBA

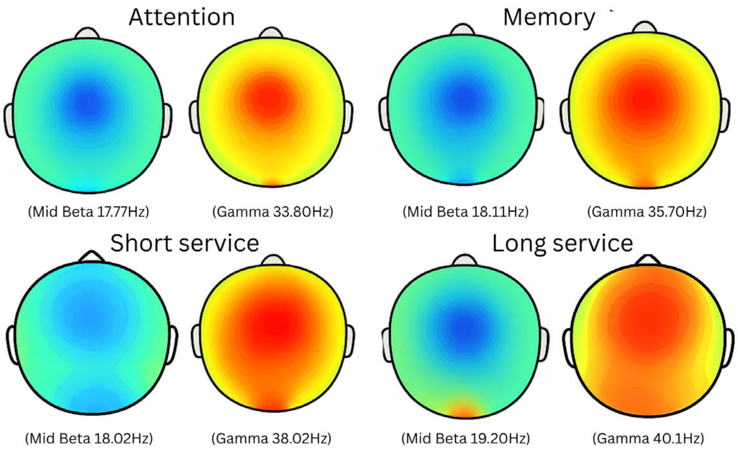

EEG spectral activity patterns in junior badminton athletes from the Life Kinetics group. Color gradients represent relative signal intensity. Numerical values indicate group-averaged dominant frequency (Hz), reflecting overall spectral characteristics of EEG activity during task performance. Left panels correspond to pre-intervention data, and right panels correspond to post-intervention data.

Descriptive EEG spectral activity patterns for the Life Kinetics group are presented Figure S1. EEG visualizations are presented for the Life Kinetics group to illustrate within-group pre–post changes in spectral characteristics across task conditions. The focus of the EEG analysis in this study is on identifying training-related trends within the intervention group. Accordingly, the presented maps highlight changes associated with the Life Kinetics intervention. The consistency of this pattern aligns with the behavioral findings, which demonstrated significant improvements in concentration, memory, and service performance following the intervention. The EEG findings are presented as exploratory indicators of relative spectral changes and should be interpreted in conjunction with the behavioral results, which provide the primary evidence for the effects of the Life Kinetics training program.

Supplementary Figure S2 EEG Brainwave Activity and Performance of IBA

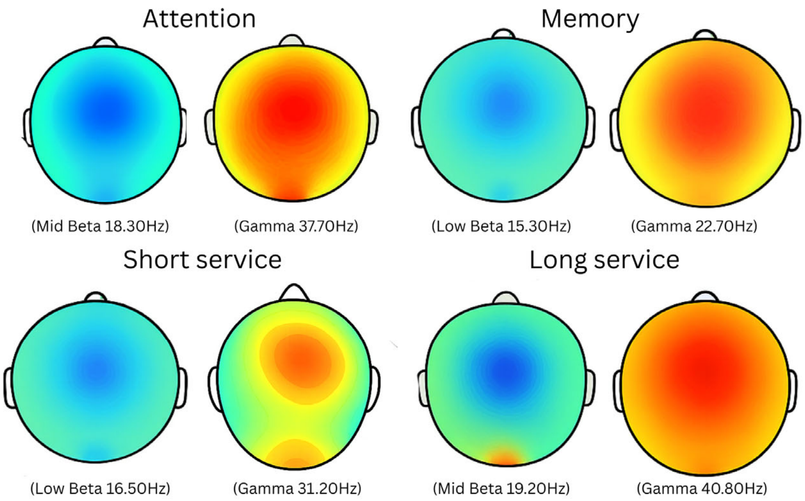

EEG spectral activity patterns in intermediate badminton athletes from the Life Kinetics group. Color gradients illustrate relative signal intensity across task conditions. Numerical values indicate dominant frequency (Hz) averaged across participants, representing spectral activity during ask performance. Pre-intervention data are shown in the left panels, and post-intervention data are shown in the right panels.

EEG spectral activity patterns indicated increased high-frequency activity following the intervention as shown in Figure S2. Similarly, EEG visualizations for the Life Kinetics group are provided to demonstrate within-group changes across conditions. The analysis emphasizes descriptive trends associated with the intervention, allowing visualization of pre–post spectral patterns during task performance. These patterns indicate increased engagement in cognitive–motor processing, particularly in tasks requiring sustained attention, working memory, and coordinated motor execution. The consistency of this pattern across multiple tasks aligns with the behavioral findings, which demonstrated significant improvements in concentration, memory, and service performance following the intervention. It is important to note that these EEG observations are presented as descriptive indicators of relative spectral changes rather than direct measures of specific neural mechanisms. Therefore, the EEG findings should be interpreted in conjunction with the behavioral results, which provide the primary evidence for the effects of the Life Kinetics training program.
